# The Dark Side of Light: Spectral Variation in Post-Traumatic Photosensitivity and Its Functional Sequelae

**DOI:** 10.3390/brainsci16080838

**Published:** 2026-08-07

**Authors:** Sandra Tosta, Geoffrey Hewitt, Adam Anderson

**Affiliations:** 1 Perceptual Development Corporation, Long Beach, CA 90808, USA; sandy@irlen.com; 2 Institute of Mathematical Sciences, Claremont Graduate University, Claremont, CA 91711, USA; geoffrey.hewitt@standardtlm.com; 3 Department of Mechanical and Aerospace Engineering, California State University Long Beach, Long Beach, CA 91711, USA; 4 Department of Psychology, Cornell University, Ithaca, NY 14853, USA

**Keywords:** photophobia, light sensitivity, head trauma, headache, migraine, neurorehabilitation, color, vision, pain

## Abstract

**Highlights:**

**What are the main findings?**

- The wavelengths of light associated with photosensitivity and head pain following mild head trauma were highly individually variable.
- Wearing lenses that successfully treated photophobia over an extended period resolved headaches and migraines as well as other associated functional complaints.

**What are the implications of the main findings?**

- Pain in response to light unlikely originated from a specific retinal receptor type with a defined wavelength range, as it was more variable, reflecting a neural hypersensitivity.
- Excessive light sensitivity contributed to headaches and migraine and associated functional impairments with its treatment having wide ranging effects.

**Abstract:**

**Background/Objectives**. Headache and migraine were exacerbated by light during an episode, with light also serving as a diathesis for pain between episodes. Such interictal photosensitivity has been hypothesized to reflect heightened sensitivity to blue-cyan (BC) light, due to activation of melanopsin-based intrinsically photosensitive retinal ganglion cells (ipRGCs). This study examined evidence for wavelength-selective photosensitivity and its relation to headaches/migraine and accompanying functional sequelae. **Methods**. Individuals with persistent post-traumatic headache (*N* = 325) were assessed for photosensitivity with an analysis of filtered wavelengths that resulted in relief. A subsample (*n* = 209) wore precision tinted lenses that matched their individual wavelength photosensitivity profile for over a 1–3-month follow up period and were assessed on a variety of persistent functional complaints. **Results**. Analysis of wavelengths associated with photosensitivity did not support differential sensitivity to the BC range, revealing greater sensitivity to light below 440 nm and less sensitivity above 680 nm, but with a coefficient of variation of 94.7%, consistent with extremely high individual variation. Individuals who showed near elimination of photosensitivity (97.9%) had decreased self-reported headache frequency (92.6%), as well as reported diminished physical, emotional and cognitive complaints, with these functional outcomes unrelated to the magnitude of BC filtering. **Conclusions**. These results suggested (1) wavelengths evoking photosensitivity were highly individually variable and (2) interictal exposure to a variety of wavelengths may have contributed to post-traumatic head pain and associated impaired global function, pointing toward common central origins.

## 1. Introduction

Abnormal light sensitivity that creates pain or photophobia has been defined as an abnormal intolerance or aversion to light that is a common symptom in a variety of neurologic conditions [1]. More recently, photophobia has been understood as a multisystem disturbance potentially involving retinal, trigeminal, thalamic, and cortical pathways, having direct overlap with pain matrix, where light regulates the neural representations of pain [2,3,4,5,6]. Beyond pain, individuals with photophobia experience nausea, visual stress and cognitive overload, even under normal lighting conditions. These complaints can persist chronically, leading to functional disability and diminished quality of life [7,8]. As one of the defining features of primary and secondary migraine [9], prevalent in traumatic brain injury (TBI) [10] and increasingly in neurodegenerative disease [11], photophobia is now known to accompany various disease states [12]. As part of a symptom cluster in various conditions, it becomes essential to understand the role of light sensitivity as a contributing or correlative factor in their expression [13]. An ongoing question, both in assessment and treatment, is whether light wavelengths differentially contribute to photophobia [14,15,16] and associated functional impairments. The question of the universal versus idiographic nature of active wavelengths in photophobia is important theoretically and in clinical practice, with the latter highlighting the potential individual diversity in the effects of color and light [17,18]. This study examined the wavelength selectivity of photosensitivity in a large cohort of individuals with headache or migraine secondary to mild traumatic brain injury. It further assessed the relationship between treatment of photophobia with adoption of spectral filters on head pain and associated functional sequelae, and the wavelength dependence of treatment effects.

Advances in sensory neuroscience, particularly the discovery of melanopsin-containing intrinsically photosensitive retinal ganglion cells (ipRGCs), have begun to describe the potential neural circuitry underlying photophobia [19,20,21]. Unlike classical rods and cones that subserve image-forming vision [22], these melanopsin-bearing retinal ganglion neurons are responsive especially to short-wavelength blue-cyan (BC), with a peak at ~480 nm, linking ambient day light to non-image-forming visual functions, including melatonin suppression, pupillary light reflex, circadian photoentrainment [23], and increasingly, photophobia [20]. Non-image-forming photoreceptors project to hypothalamic, thalamic, and limbic structures involved in pain and affective processing, establishing anatomical and functional links between light detection and pain and aversion pathways [24,25], transforming light into discomfort or nociceptive percepts, particularly in sensitized states characterized by photophobia, such as migraine and TBI [12].

Wavelength selectivity of photophobia would then originate from hypersensitivity of neural circuits that receive information from non-image-forming photoreceptors, which critically assess irradiance and use brightness to regulate primitive, but essential, light regulatory responses [26,27]. Rather than visual perception of specific colors as being aggravating, which would involve classical photoreceptors, i.e., rods and cones along the geniculostriate path that supports image-forming perceptual awareness, a narrow band of wavelengths regulates both a perception of brightness and pain. An adaptive response to brightness would then be rendered maladaptive in individuals with extreme photosensitivity. Complicating this simple dissociation, rods and cones also contribute to brightness perception and regulate ipRGCs, and ipRGCs modulating the image forming pathway [28,29] and the modulation of photophobia [16]. There is evidence that a broad array of wavelengths can regulate neural responses in light-regulating functions that were thought to be selectively modulated by ipRGC’s, such as human circadian clock, with substantial individual variability and context specificity of narrow band light sensitivity [6,30,31]. These interactions, coupled with emerging evidence of inter-individual variability, suggest that photophobia may not reflect a single mechanism, but a family of hypersensitivity syndromes rooted in multiple pathways, whereby image-forming and non-image-forming light sensing modes are combined in the brain. This would add significant complexity to characterizing photophobia phenotypes and their wavelength dependency.

These complex interactions likely continue and are amplified in the brain, with evidence from a variety of sources showing that the different wavelengths of light can regulate brain activity well beyond circadian rhythms [32], including visual cortical activity [33,34,35], motivation and learning [36], and high-level cognition [37]. Within these color modulatory effects there are potent individual differences in responsiveness to, and interactions between, different colors, even in those without heightened photosensitivity [18]. Color may regulate midbrain reward and pain centers, viscerosensory interoceptive cortex, and associated autonomic activity, as well as the functional connectivity across brain regions [36]. Such large-scale networks altered by incident wavelengths provide a background context for significant heterogeneity of effects of light. Even the most peripheral downstream sources of photophobic pathophysiology of ocular origin [2], including dry eye, ocular inflammation, and corneal disorders, could amplify these photosensitive neural processes from non-image-forming ipRGCs or image-forming pathways. 

In the most common neurological context, headache and migraine, photophobia has been reported in 80% of individuals during an episode [38]. Headache is a common and enduring concern in patients with traumatic brain injury [39] that is often experienced interictally [40], between headache episodes, which contributes to disability and may represent a diathesis for other associated functional impairments. Photophobia and headache/migraine, often occur following even mild TBI [12,41], and serve as a diagnostic for secondary headaches of TBI origin [42], which may persist for years [43,44,45,46,47]. When chronic and unremitting to standard treatments, these issues can be debilitating, with persistent difficulties beyond color and light sensitivity [48], such as issues with perception, attention and concentration [12,49], resulting in documented academic or workplace struggles [50]. Standard optometric and ophthalmological care and treatment do not always relieve photophobic symptoms [51], with persistent photophobia associated with continued neural dysregulation and sensitization to light [52]. As such, photophobia in TBI often represents as a central sensitization, with light having the capacity for dysregulation of neural activity at the intersection between the visual and pain pathways [53], which is regulated by ipRGC inputs [15], but likely not fully explained by them [54].

The present study examined visual discomfort to light in active-duty military or veterans who experience medically resistant post-traumatic headache or migraine. Photophobia after TBI and concussion is routinely addressed with sunglasses and precision-tinted colored lenses to alleviate symptoms [46,55,56,57,58]. This study set out to index the commonality and variability of light wavelength sensitivity in this common treatment modality. In a large sample, a subtractive method was used to index selectivity and variability in wavelength, whereby participants were examined under various combinations and saturations of color filtering [59,60] to determine which best diminished light discomfort and associated visual stress symptoms. After adopting lenses that resolved photophobia, post-traumatic headache and migraine symptoms, and other physical and cognitive symptoms, were further tracked. The study examined whether photophobic response to light reflected a common differential sensitivity to the BC band, compared to other wavelength bands, and whether the BC band predicted subsequent resolution of physical and cognitive sequelae associated with post-traumatic headache. The alternative hypotheses were whether an overall reduction in light intensity or an idiographic differential sensitivity to light in wavelength bands was more characteristic of post-traumatic photophobia, headache and migraine, and associated functional sequelae.

## 2. Materials and Methods

### 2.1. Sample

The study employed a secondary analysis of data gathered as part of a private clinical exam based on a referral from a doctor for the use of spectral filter lenses to address persistent light sensitivity, headaches and migraines after TBI and standard military medical acute and recovery care, including optometric and ophthalmologic care. Participants were not specifically recruited to participate in research. Informed consent was obtained as part of a broader clinical protocol gathered during the initial intake appointment, requesting future use of clinical data for research purposes.

The study investigated 325 individuals that were current or previously active military servicemen and women (97% men) who had sustained TBI or concussion (M = 2.88 *SD* = 2.99 years prior) associated with light sensitivity, chronic headaches or migraines (see Table 1), who were referred for evaluation and treatment, with a final subsample of 209 who completed all measures again post treatment of photophobia with spectral filter adoption. Data were collected during a standard clinical exam after referral from a doctor or associated healthcare provider for potential treatment of visual symptoms with spectral filters. Clinical screening was not modified to accommodate the present research. 

All participants reported “medically resistant” headaches or migraines, as they had not responded to standard courses of treatment (see Table 1). It was at the doctor’s discretion to assess light sensitivity and visual stress, and to indicate the use of spectral filters and conduct follow-up examinations. Participants reported a variety of other behavioral, emotional, physical, performance-related, and visual–perceptual processing symptoms often associated with TBI and PTSD [39,41,42,45,61].

Consent was obtained for permission to use diagnostic responses for research purposes. Personal identifiers were separated from questionnaire data and from therapeutic files. The study was reviewed and verified as Exempt according to 45CFR46.104(d)(4): (4) Secondary Research Uses of Data or Specimens by Solutions Institutional Review Board (IRB Registration #: IORG0007116, Federal wide Assurance (FWA) #: IRB00008523).

### 2.2. Procedures

Participants answered a questionnaire assessing headache and migraine frequency (per day and per month), medication usage, and difficulties associated with multiple functional areas. They took part in a two-part diagnostic process for a more complete history of their concerns and symptoms [59] and to determine wavelengths that resulted in maximum self-reported light sensitivity and visual stress relief [60]. The two parts were completed immediately after each other. The result was the generation of individualized photophobia profiles that were employed to generate precision-tinted spectral filters worn as glasses to attenuate specific wavelengths of light related to difficulties for the individual as revealed during the diagnostic procedure. For the purposes of illustration, the unique light sensitivities relative to a reference commercial standard, e.g., FL-41, optical tints, limiting transmittance in the blue-cyan (BC) range between 440 and 550, maximally at 480 nm, and the peak sensitivity for ipRGC were examined. 

Individuals were asked to wear their glasses daily. Adherence was not assessed during follow-up. Questionnaires were administered over the phone 4–12 weeks later. This 1–3 month period depended on scheduling availability. During this follow-up, pre-intervention assessments were repeated.

### 2.3. Instruments

Multiple Function Questionnaire: To ascertain potential areas of concern, a custom questionnaire assessing 32 subareas was administered. Beyond assessing light sensitivity and headache severity, areas of concern included attention and concentration, academic/job performance, emotional symptoms, physical symptoms, vestibular-related items such as balance, and sleep quality. Difficulties were rated on a 0–5 scale, 0 indicating “no problem,” and 5 indicating “considerable problem” [60]. 

Wavelength filtering: To achieve maximum light sensitivity reduction and improvement in visual stress, the aggravating wavelengths were identified using a structured diagnostic process [59,60]. During the diagnostic protocol, a set of 65 filters, varying in hue and transparency, was systematically combined to create an individualized spectral filter. This custom color curve was applied to either plano (non-prescription) or prescription optical lenses in an optical tinting lab and worn as glasses. The protocol included activities to stress the visual system (e.g., light tolerance, letter and word reading, tracking moving objects and across lines of text) to delete wavelengths associated with discomfort and reported symptoms when doing visually intensive activities. Subjective performance on the same tasks was assessed with and without spectral filters. In addition to characterizing wavelength sensitivity, this resulted in a unique color formula for each individual characterized by a spectrophotometer-determined spectral curve and then tinted on lenses to be worn as glasses, i.e., the precision-tinted spectral filters. Validity testing of the diagnostic test manual has been conducted, and a high reliability of color choice has been identified for spectral filter determination [62,63]. The spectral curves from the spectrophotometer were analyzed to examine potential wavelength specificity of photophobia.

### 2.4. Data Analysis

Wavelength specificity of light sensitivity. To investigate wavelength-specific sensitivities of light sensitivity in photophobia and the potential of corresponding improvements in migraine frequency and migraine severity following photophobia treatment, data were analyzed using a series of hierarchical linear regressions for examination of whether the spectral filter conditions and baseline variables (light sensitivity, migraine frequency, migraine severity) predicted post-intervention outcomes for migraine frequency, migraine severity, or light sensitivity. In the first step (M_0_), an intercept-only model was estimated to provide a baseline level of unexplained variance. In the second model (M_1_), the percent of light transmission at each spectral variable in 40 nm bins (400, 440, 480, 520, 560, 600, 640, 680) and appropriate baseline measurement (PRE score) were entered to evaluate their unique and combined contributions to post-intervention outcomes for migraine frequency, severity, and light sensitivity. Model fit was assessed using R, R^2^, adjusted R^2^, change in R^2^, RMSE, and the significance of the R^2^ change. Regression coefficients were examined to determine the magnitude and direction of associations between each predictor and the outcome. All analyses were conducted using standard assumptions of linear regression, including examination of residual distributions and multicollinearity diagnostics. Analyses were conducted with JASP Team, 2022 [64] Version 0.16.3.

To further quantify the degree of spectral similarity among individualized spectral filters, a pairwise Euclidean distance analysis was conducted on a larger set of lens transmission curves from 325 individuals with photophobia and medically intractable headache or migraine. Each spectral filter lens was represented as a vector of transmission values across the measured wavelength spectrum, and Euclidean distances were computed between every possible pair to assess the magnitude of spectral dissimilarity. This approach provides a continuous measure of how distinct any two filters are in terms of their spectral profiles, with larger values indicating greater divergence in light-transmission characteristics. Descriptive statistics (minimum, maximum, mean) were calculated to summarize the overall distribution of distances, and a coefficient of variation (CV) was computed to assess relative variability in spectral differences across the full set of spectral filter lenses. This analytic strategy allowed for a quantitative evaluation of the extent of individualization present in the dataset. 

Multi-Domain Function. An exploratory correlation analysis of the multiple function questionnaire confirmed that responses within a subarea were highly correlated. We chose the most generally worded item from each area for further analysis. We then grouped symptoms into seven factors based on observed construct association and conducted a confirmatory factor analysis (CFA) using maximum likelihood estimation to evaluate the hypothesized seven-factor model of symptom domains. We submitted pre- and post-intervention scale data for subareas to multivariate analysis of variance (MANOVA). Follow-up univariate analysis was employed to assess effect sizes. To address skewedness in the scale data, we employed Wilcoxon Signed-Ranks Tests with Bonferroni correction for significance testing. Wilcoxon Signed-Ranks Tests with Bonferroni correction were used to investigate changes in migraine frequency and medication use. All tests were two-tailed, and the statistical significance level was set at *p* ≤ 0.05. 

## 3. Results

Light Sensitivity. Consistent with the selection criteria for photophobia, participants’ largest concern was their light sensitivity (Table 2), self-reported as severe, 4.8, *SD* = 0.42. The hypothesis was that light sensitivity is specifically related to central pain. Pearson correlation coefficients were computed to assess the linear relationship between light sensitivity and headache and migraine. 

**Figure 1:**
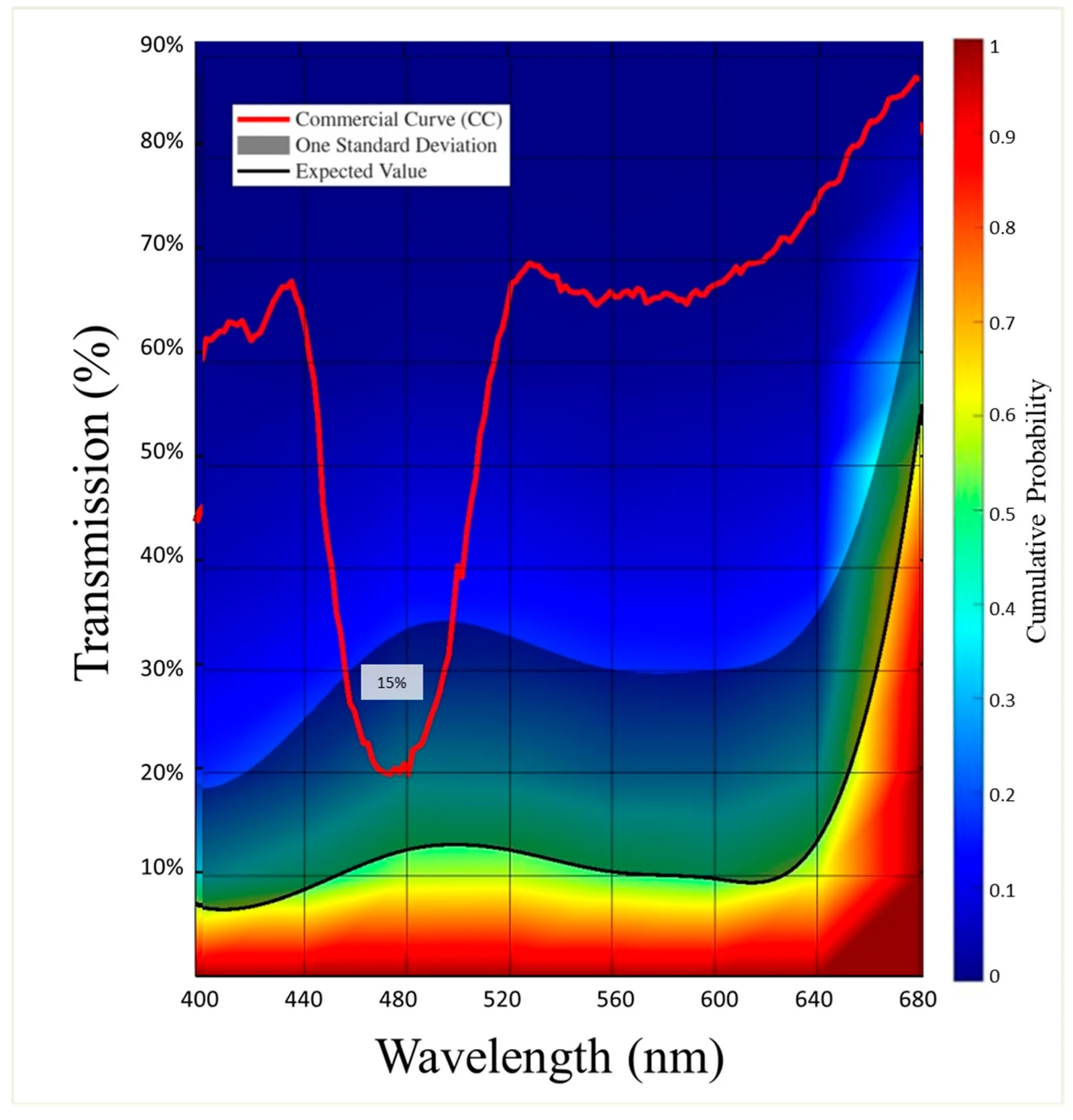
Percent of light transmission by wavelength band. Black line represents the average light transmission to resolve photosensitivity, with the shaded area representing +1 Standard Deviation. The color range illustrates cumulative probability of transmission within the respective wavelength bin. For reference, the red line indicates commercial (Fl-41) notch filter transmission, which should selectively attenuate melanopsin-based intrinsically photosensitive cells, representing 15% of participants reported photosensitivity.

Spectral Filter Color Distribution. The individualized optimal filter color and transparency contained a combination of several colors, filtering across the entire visual spectrum to varying degrees. Beyond a common reduction in transmittance, pairwise Euclidean distance analysis of filtered wavelengths revealed a wide range of spectral dissimilarity (minimum = 0.74, maximum = 224.00, mean = 61.00). The coefficient of variation (94.7%) indicated extreme heterogeneity in wavelength filtering, for optimal relief. In Figure 1, the bold black line represents the average transmission in each bin. This line is essentially flat, except for a decrease in filtering at the longest wavelength bands (600–680). The commercial FL-41 lens (red line) that aligns most closely with BC melanopsin-based filtration, with peak filtering around 480 nm, meets the needs of 15% of the sample.

**Figure 2:**
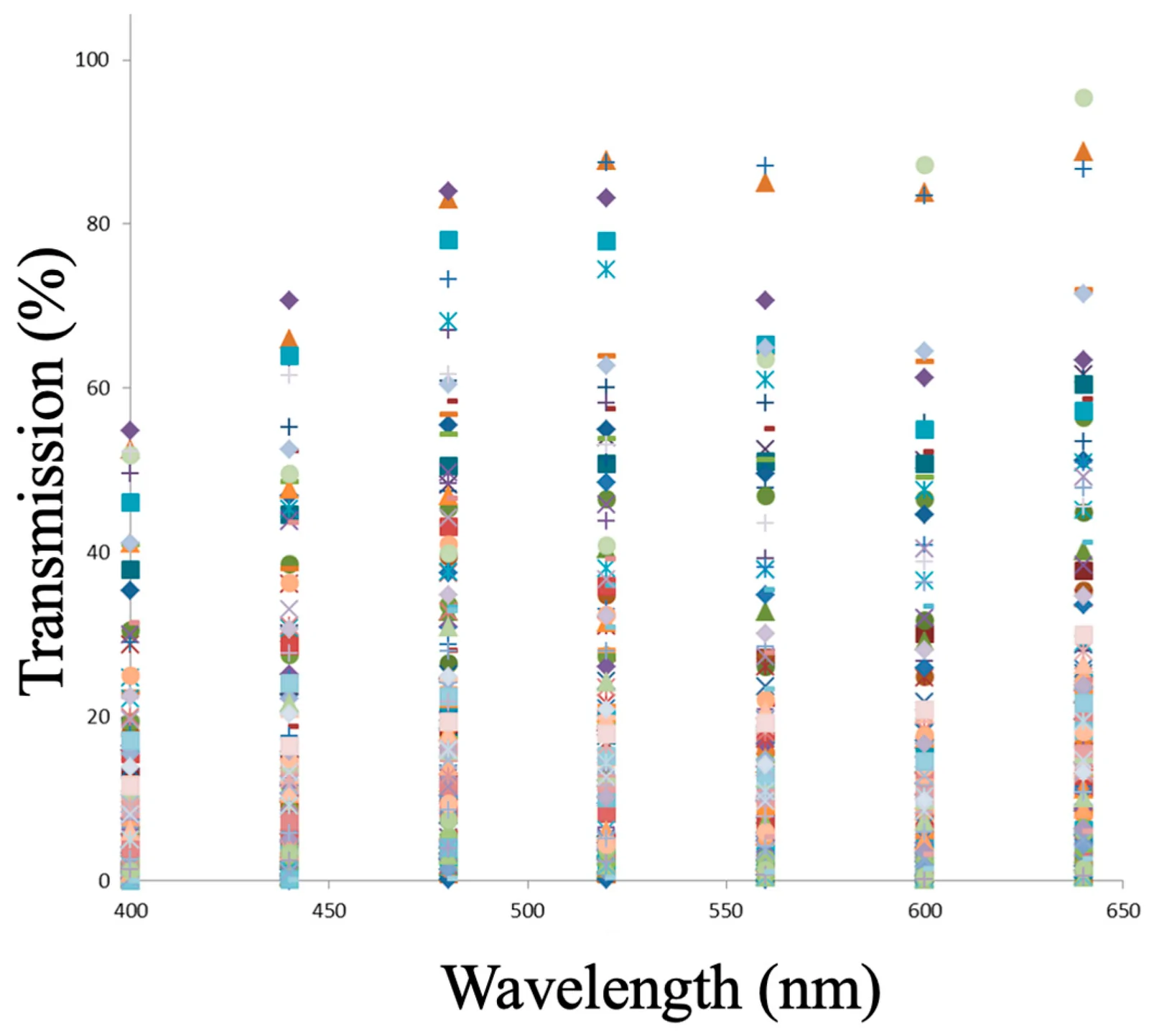
Scatterplot of individual filtering by wavelength band. The coefficient of variation (94.7%) of pairwise Euclidean distance analysis of filtered wavelengths indicates extreme heterogeneity. Each symbol represents a unique individual.

A univariate ANOVA was conducted to examine the effect of spectral band on light transmission. Due to violation of homogeneity of variance (Levene’s *F* = 4.24, *p* < 0.001), Welch’s ANOVA was conducted. A significant main effect of the spectral band was found for light transmission, *F*(7, 1200) = 236.30, *p* < 0.001. Given the significant effect of spectral band on light transmission, post hoc pairwise comparisons were conducted using Tukey’s HSD test to examine differences between spectral bands. Descriptive statistics revealed that 680 nm (*M* = 77.62, *SD* = 13.40) had substantially higher transmission values compared to all other bands, which ranged from *M* = 10.28 (*SD* = 12.94) for 400 nm to *M* = 20.25 (*SD* = 19.32) for 640 nm. Among wavelengths 400 nm through 640 nm, several significant differences emerged. The 400 nm wavelength showed significantly lower transmission than wavelengths 480, 520, 560, and 640 (all *ps* < 0.05). Furthermore, 440 nm demonstrated significantly lower transmission than 480 nm (*p* < 0.001) and 640 nm (*p* = 0.012). No other pairwise comparisons among wavelengths 400 nm through 640 nm reached statistical significance after correction for multiple comparisons. A scatter plot, Figure 2, further illustrated the spread of transmission levels in each wavelength bin, highlighting the individualized aspect of light sensitivity across the entire visible spectrum.

### 3.1. Self-Report Ratings in Critical Areas of Function

To reduce item-level measurement error and capture the latent structure underlying symptom responses, individual questionnaire items were aggregated into construct-based factors representing theoretically derived symptom domains. This dimensional approach facilitated a more parsimonious representation of the data and provided construct validity for subsequent analyses. A confirmatory factor analysis (CFA) using maximum likelihood estimation evaluated the fit of a hypothesized seven-factor measurement model, reflecting the latent domains of somatic discomfort, vestibular/balance difficulties, visual performance tasks, perceptual and driving challenges, emotional regulation, cognitive processing, and fatigue/sleep symptoms. Model fit was assessed using multiple indices, including the chi-square statistic, comparative fit index (CFI), Tucker–Lewis index (TLI), incremental fit index (IFI), root mean square error of approximation (RMSEA) with 90% confidence intervals, standardized root mean square residual (SRMR), and information criteria (AIC, BIC, and sample-size adjusted BIC). Factor loadings, variances, and covariances were examined to evaluate the internal structure and discriminant validity of the latent constructs.

The hypothesized seven-factor model demonstrated a statistically significant chi-square value, χ^2^(384) = 690.22, *p* < 0.001, which is expected in large samples given the test’s sensitivity to sample size. Comparison with the baseline model, χ^2^(435) = 2211.65, indicated a substantial improvement in model fit. Overall, the model demonstrated acceptable fit to the data. The comparative fit index (CFI = 0.828), Tucker–Lewis index (TLI = 0.805), and incremental fit index (IFI = 0.832) approached conventional thresholds for adequate fit (>0.90), suggesting a reasonable, though improvable, representation of the observed data. The root mean square error of approximation (RMSEA = 0.062, 90% CI [0.054, 0.069]) fell within the acceptable range (<0.08), with a significant test of close fit (*p* = 0.005). The standardized root mean square residual (SRMR = 0.075) indicated acceptable residual covariance, while the normed fit index (NFI = 0.688) and relative fit index (RFI = 0.646) fell below ideal cutoffs, suggesting some potential for model refinement. Information criteria were as follows: AIC = 18,570.20, BIC = 18,941.20, and sample-size adjusted BIC = 18,589.50, with a log-likelihood of –9174.10 and 111 free parameters. Hoelter’s critical *N* indicated adequate model stability for samples exceeding approximately 131 participants at α = 0.05.

All observed indicators loaded significantly on their intended latent constructs (*ps* < 0.01), supporting the hypothesized structure. Factor 1 (Somatic Discomfort) included Headaches, Migraines, Eye strain/pain, Nausea, and Stomachaches (standardized loadings = 1.07–4.41, *ps* < 0.01). Factor 2 (Vestibular/Balance Difficulties) was defined by Dizziness, Tracking moving objects, Coordination, Balance, and Depth perception (1.12–1.52, *ps* < 0.001). Factor 3 (Visual Performance Tasks) comprised Reading, Computer use, Job performance, Copying, Math computation, and Paper and pencil tasks (1.67–4.93, *ps* < 0.001). Factor 4 (Perceptual and Driving Challenges) included Driving, Night driving, Watching TV or movies, and General perception (0.32–0.95, *ps* < 0.001). Factor 5 (Emotional Regulation) was reflected by Behavior control/anger, Agitated/irritable, Anxious, Depressed, and Fidgety (0.71–1.26, *ps* < 0.001). Factor 6 (Cognitive Processing) included Poor concentration, Slowed thinking, and Short-term memory loss (0.95–1.93, *ps* < 0.001). Factor 7 (Fatigue/Sleep) comprised Tired/fatigued and Sleep problems (0.61, *p* < 0.001). All factor variances were significant (*ps* < 0.05), indicating meaningful variability within each latent construct. Inter-factor covariances ranged from 0.04 to 0.64 (*ps* < 0.01), suggesting that while the factors were distinct, they were moderately correlated and reflected related dimensions of visual, perceptual, and affective functioning. Residual variances for individual indicators were all significant (*ps* < 0.001), indicating that each observed variable contained both shared and unique variance not fully explained by its latent factor.

Taken together, the CFA supported the hypothesized seven-factor measurement model, demonstrating a coherent and interpretable latent structure. Model fit indices indicated an acceptable approximation of the data, providing empirical support for the distinct yet related symptom domains. These findings validate the aggregation of individual questionnaire items into construct-based factors and justify their use in subsequent analyses examining relationships among visual–perceptual, cognitive, and affective dimensions.

### 3.2. Symptom Change Following Spectral Filter Adoption

#### 3.2.1. Light Sensitivity 

As designed, wearing spectral filters nearly eliminated light sensitivity, with 94% rating it a 0 (out of 5), and 99.5% rating it 0–2 (out of 5). This change was assessed as part of a multiple analysis of variance (MANOVA), (*F*(1, 357) = 16031, ηp2 = 0.98), Wilcoxon Signed-Ranks (*z* = 11.95, *p* < 0.001), representing the area of largest intervention effect size. 

A linear regression was conducted to examine whether baseline light sensitivity predicted post-intervention light sensitivity. The model was not significant, (*F*(1, 151) = 0.01, *p* = 0.945), and explained no variance (R^2^ < 0.001). No further models were examined because no filter variables were associated with light sensitivity.

#### 3.2.2. Migraines

Participants reported experiencing significantly fewer migraines per month (M = 1.8, *SD* = 7.2) following spectral filter adoption, (*z* = 11.23, *p* < 0.001), with 76% of the sample no longer reporting migraines. In addition to self-reported changes in the magnitude and frequency of symptoms, wearing spectral filters was associated with a significant decrease in the use of prescription medication (Rx) (*z* = 9.30, *p* < 0.001) and over-the-counter (OTC) (*z* = 7.12, *p* < 0.001) medications, resulting in 74% reduction in Rx and 99% reduction in OTC medicine usage.

#### 3.2.3. Physical, Vestibular, Academic, Environmental Processing, Emotional, Cognitive, and Sleep

A one-way repeated measures MANOVA was conducted to examine changes in symptom severity across eight concussion-related domains from pre- to post-intervention. The multivariate test using Pillai’s trace revealed a statistically significant time effect, *V* = 0.974, (*F*(8, 350) = 1652, *p* < 0.001), indicating substantial overall improvement across the symptom domains following adoption of color filters. To further examine the nature of these changes, a series of Wilcoxon Signed-Ranks tests were conducted to assess pre- to post-intervention differences across the eight individual domains: Physical, Vestibular, Academic, Environmental Processing, Emotional, Cognitive, Sleep, and Light Sensitivity. Given the use of multiple comparisons, a Bonferroni correction was applied, yielding an adjusted alpha level of 0.00625 (α = 0.05/8).

Results indicated statistically significant improvements across all eight domains, with all *p*-values < 0.001, remaining significant even after the conservative Bonferroni correction. Effect sizes, calculated as *r* = *z*/√*N*, were exceptionally large across all domains. In addition to the Light Sensitivity (*z* = 11.95, *r* = 1.00), with the greatest mean change post filter adoption, the Physical domain showed significant improvement (*z* = 11.92, *r* = 1.00), as did the Vestibular (*z* = 11.88, *r* = 1.00), Academic (*z* = 11.89, *r* = 1.00), Environmental Processing (*z* = 12.02, *r* = 1.00), Emotional (*z* = 11.51, *r* = 1.00), Cognitive (*z* = 11.17, *r* = 1.00), and Sleep (*z* = 10.09, *r* = 0.98) domains. The spectral filter intervention for photophobia did impact some domains less successfully than others. Spectral filters were most effective for reducing light sensitivity; challenges with environmental visual processing; academic and workplace performance issues; vestibular and balance difficulties; and somatic discomfort in the head, eyes, and stomach, with mean post scores for all these domains <0.50 (out of 5). In contrast, participants reported continued moderate challenges after intervention in the areas of emotional regulation, cognitive processing, and sleep/fatigue, all having post means > 1.80 (out of 5). 

#### 3.2.4. Role of Time Since Injury 

Given the variation in the length of time since injury in the sample, an exploratory analysis was conducted to address the potential differential influence in response to adoption of spectral filter lenses, beginning with the most pronounced functional sequalae for seeking treatment: migraines. A Gamma-distributed generalized linear model, to correct for normality violations, was fit predicting post-treatment migraine frequency from baseline migraine frequency and years since injury. While migraine pre-score was a significant predictor of the size of the intervention effect, with greater pre-intervention frequency associated with greater improvements (*z* = 3.27. *p* = 0.001), years since TBI diagnosis was not significant (*z* = −0.028, *p* = 0.239). The intervention was effective at reducing migraine frequency whether the injury was less than one year, 1–5 years, or more than 5 years prior to intervention, with more than 70% of each group reporting no migraines after intervention. Similarly, years since TBI diagnosis did not predict the intervention effect for migraine severity (*p* = 0.369) or light sensitivity (*p* = 0.252).

### 3.3. Wavelength-Specific Impact on Light Sensitivity, Migraine Severity and Frequency

A one-way multivariate analysis of variance (MANOVA) was conducted to examine the effect of spectral band on multiple outcome variables, including light transmission, migraines per month, migraine symptoms, and light sensitivity post-injury. The MANOVA revealed a statistically significant multivariate effect of spectral band, Pillai’s trace = 0.586, *F*(28, 4800) = 29.41, *p* < 0.001, indicating that spectral band had a significant overall effect on the combined dependent variables. A series of linear regression analyses were then conducted to examine the relationships between various wavelengths of light exposure and symptoms, as well as the relationships between pre- and post-filter adoption symptom measures.

Overall, the percent of light transmission across eight key 40 nm spectral bands did not reliably predict or explain differences in post-adoption symptoms. While percent light transmission at 520 nm emerged as a significant predictor, in the model, (*b* = −0.011, *SE* = 0.005, β = −0.183, *p* = 0.023), the model explained only 3.4% of the variance in migraine symptoms (*R*^2^ = 0.034), and the direction of influence was the opposite of decreased transmittance. Consistent with green light as prophylactic against migraine [65], higher 520 bin values (i.e., greater light transmission) were associated with decreased migraine severity. For frequency of migraines per month, a model including transmission at 680 nm was negatively associated with post migraine frequency (*b* = −0.049, *SE* = 0.023, β = −0.171, *p* = 0.038), with higher light transmission predicting lower frequency, explaining 2.9% of the variance (*R*^2^ = 0.029). 

## 4. Discussion

In individuals with post-traumatic headache or migraine who also experience photophobia, there was little evidence to support theories of common aggravation from specific wavelength bands of light and specifically those theorized to be most problematic in the blue-cyan (BC) range that maximally activate melanopsin-based intrinsically photoreceptive retinal ganglion cells (ipRGCs). There was evidence of common filtering of short relative to long wavelengths, but more consistently there was a highly individually variable pattern, consistent with an idiographic, rather than universal, basis of wavelength selectivity in photophobia. An intervention study that applied these specific individually defined wavelengths to spectral filter lenses worn for 1–3 months abated photophobia and the intensity and frequency of headache and migraine and secondarily diminished various domains of associated complex functional symptoms. The amount of filtering in the BC range did not relate to these outcomes but there was evidence consistent with prior findings that transmittance of green light is prophylactic, associated here with decreased migraine intensity and anxiety.

A traditional and commonsense view would have photophobia related to the overall intensity of light. As shown here, there is substantial evidence that wavelengths of light, rather than, or in addition to, overall intensity, characterize light sensitivity at the level of individuals. In migraine populations, blue light is thought to preferentially drive discomfort [66,67]. The present results demonstrated that the wavelengths that elicited discomfort varied substantially across individuals and did not cluster around melanopsin’s spectral peak. This lack of convergence is difficult to reconcile with a strictly ipRGC-centric model, which would predict relatively consistent spectral sensitivity profiles across individuals based on conserved photoreceptor physiology [22]. This study approached an understanding of this individual variability in light wavelength-generated pain from changes in the brain downstream of the retina [6], particularly in the present sample of individuals with post-traumatic headache and migraine. If photophobia arises from central amplification mechanisms, then variability in perceived discomfort across wavelengths could reflect individual differences in how visual signals are weighted and interpreted at the thalamic or cortical level, beyond differences in retinal encoding [15].

The most salient feature of the current findings was the marked inter-individual variability in wavelength sensitivity. Under a retinal model, such variability would be unexpected, as spectral sensitivity functions are relatively invariant across individuals. If photophobia reflects altered gain control within distributed neural circuits, then individuals may develop distinct spectral trigger profiles depending on how visual input interacts with nociceptive and salience networks [68,69]. This interpretation is consistent with evidence that photophobia varies widely across individuals even within the same diagnostic category [47]. Neurochemical modulators may further contribute to this heterogeneity. For example, calcitonin gene-related peptide (CGRP) has been implicated in both migraine and TBI-related sensory hypersensitivity and is known to enhance neuronal excitability within trigeminothalamic circuits [41]. Variability in neuromodulatory systems could produce person-specific amplification of different spectral inputs, rather than a uniform shift toward short-wavelength sensitivity. In the context of post-traumatic headache, this central model is particularly compelling. Traumatic injury produces heterogeneous alterations in thalamic function, cortical excitability, and large-scale network connectivity, all of which are known to influence sensory gain and salience processing [46].

The relationship between photophobia, headache/migraine, and cognitive function is complex and multidirectional. Photophobia can be both a symptom and a cause of central dysregulation. Drugs that decrease migraine also attenuate photophobia [70]. Similarly, spectral filter lenses resolve photophobia, regulate headache and migraine, and attenuate many physical, mental and cognitive disturbances following head injury [13], as assessed in the present findings. Retinal ganglion cells modulate the thalamic relays of the trigeminovascular nociceptive meningeal pathway to the somatosensory cortex, with these same thalamic neurons projecting to the visual cortices [20], which may further support visual cortical hyperexcitability [71,72]. The pathways supporting photophobia are also implicated in migraine itself: the ictal condition exacerbates sensitivity to light, while exaggerated interictal light sensitivity, in turn, serves as a trigger for migraine [14,15]. This shared circuitry, organized through recurrent thalamocortical connections, could become self-reinforcing through positive feedback, in which pain influences light sensitivity and light sensitivity influences pain, with effects reverberating across multiple levels of the nervous system. This feedback loop has been thought to produce diverse, widespread patterns of dysregulation, ranging from hypothalamic to high-level cortical involvement [73], and spanning autonomic, affective, and cognitive domains [13].

Photophobia may fundamentally be a disorder of central sensory integration, involving convergence between visual and nociceptive pathways [5]. Experimental and clinical studies have demonstrated that light signals can activate nociceptive circuits via the trigeminothalamic pathway, providing a substrate through which light is experienced as painful [4,41]. These pathways are thought to be sensitized in conditions such as migraine and TBI, leading to increased gain and lowered thresholds [74,75] for discomfort. While melanopsin-driven input contributes to sustained discomfort responses, these responses are likely modulated by higher-order processing and context-dependent factors, rather than determined solely by a restricted wavelength band. Recent evidence suggests individual differences in susceptibility to color-induced visual discomfort even in normal function [18]. Individuals susceptible to pattern glare, sensitivity and discomfort to repeated high contrast lines, which is associated with exaggerated visual cortical activity or hypersensitivity, experienced more discomfort to light that differed in color distance [18]. Discomfort was not predicted by models of contrastive cone coding, but rather cortical representations of color. Consistent with normative differences in color coding across individuals [36], this supported the interpretation that central processing—more so than retinal tuning—governed the subjective experience of photophobia, which could be further heightened in migraine and following head trauma [6,76]. It also may provide an account for why individual photophobia expression can differ in the color of light that creates discomfort and its pathological amplification.

These findings have important implications for both mechanistic models and clinical practice. First, they suggest that standardized wavelength-targeted interventions, such as blue-light filtering lenses, such as Fl-41, may be insufficient for many patients, or induce greater pain in some individuals. While such approaches are grounded in melanopsin biology and can be effective in some populations [12], their inconsistent efficacy may reflect a mismatch between retinally targeted interventions and centrally mediated dysfunction. As the current data suggest, such interventions may worsen other sequelae associated with photophobia of post-traumatic origin. The present findings highlight the importance of developing objective biomarkers of central sensory processing, such as neuroimaging or electrophysiological measures, to better characterize whether individual variation in the spectral profile of photophobia originates from a common dysregulation, or an idiographic central dysfunction. Such approaches may help identify the neural correlates of individualized spectral sensitivity and guide targeted interventions.

In terms of limitations, the study used a method of subtraction, removing wavelengths that created discomfort. While this is more amenable in practice to a variety of testing conditions without need for complex equipment, its outcomes may differ from additive methods. While this method has been shown to result in reliable indication of highly individually variable color filters [62,63], this test–retest reliability was not assessed in the present large sample. It is also limited by a sample that is almost entirely of men and who expressed neurological concerns beyond photophobia and headache/migraine. While the sample received a complete clinical evaluation prior to participating, including optometric and ophthalmologic services, the role of these concerns as they relate to photophobia and the spectral filter intervention are unknown and warrant further investigation. Previous research has suggested that ocular motor dysfunction is common post TBI, and can contribute to continued challenges with optical focus, tracking, and binocular alignment, and can respond positively to neuro-optometric interventions allowing for comprehensive post-TBI care [77]. 

More broadly, these findings thus may not generalize to other etiologies of photophobia. In this sample, although photophobia was the most salient concern, larger central nervous system dysregulation is very likely their cause of photophobia. Other forms of photophobia with concomitant functional disturbances may have a different profile more consistent with melanopsin-mediated blue light sensitivity. While the spectral content of filters did vary substantially on an individual basis, they reduce overall light exposure in addition to selectively blocking specific wavelengths, making it challenging to disentangle intensity and spectrum effects. However, individuals with greater transmittance also did not demonstrate a common greater filtering of wavelengths in the ipRGC range. An active control condition that lessens transmittance of all wavelengths, i.e., sunglasses, or those with a standard notch filter, such as FL-41 lenses, would be an important improvement in experimental design for treatment of photophobia and associated sequelae. Overall, and consistent with recent arguments for a more idiographic approach to understanding light regulation of vision and health [17], more work is needed to understand the origins of the effects of light, in illness and health, at the level of individuals.

## 5. Conclusions

In summary, while melanopsin and ipRGC pathways contribute to photophobia, evidence of observed heterogeneity in photophobic wavelength sensitivity indicates that retinal mechanisms are insufficient to account for its expression. The absence of a consistent spectral profile in post-traumatic photophobia supports a model in which photophobia is understood as a centrally mediated disorder of sensory gain and integration, in which the same retinal inputs, depending on the individual, are differentially transformed into central pain signals. This framework suggests individual variability is essential to consider in the development of personalized diagnostic and therapeutic strategies for photophobia and its functional sequelae. Objective spectral phenotyping may show promise for matching patients to the best optical or neuromodulatory therapy.

## Figures and Tables

**Table 1:** Demographics.

	Sample	
	n	%
Gender		
Male	202	97
Female	7	3
PTSD diagnosis	138	66
	M (SD)	Range
Age in years	30.26 (6.48)	20–49
Years since TBI diagnosis	2.88 (2.99)	0–15
Migraines/month	24.08 (26.50)	0–126
Rx doses/month	66.49 (73.94)	0–370
OTC doses/month	31.73 (70.31)	0–455

**Table 2:** Self-reported concerns in light sensitivity and other functional domains pre and post 1–3 months of adopting spectral filters. (5 = considerable concern).

	PRE		POST		
	Mean	Std. Deviation	Mean	Std. Deviation	Mean Change
Factor 1 Physical	3.58	0.84	0.40	0.77	3.18
Factor 2 Vestibular	2.91	1.33	0.38	0.69	2.53
Factor 3 Academic	3.92	0.88	0.22	0.49	3.70
Factor 4 Environmental Processing	3.86	0.94	0.10	0.30	3.76
Factor 5 Emotional	4.02	0.97	1.82	1.36	2.20
Factor 6 Cognitive	4.20	0.84	1.91	1.34	2.30
Factor 7 Sleep	4.47	0.80	2.73	1.73	1.74
Light Sensitivity	4.80	0.42	0.10	0.43	4.7

## Data Availability

The data presented in this study are available in Figshare at https://figshare.com/articles/dataset/Spectral_Variation_in_Post_Traumatic_Photosensitivity_Lens_Transmission_and_Primary_Subject_Data_Sets/32736117?file=65692149 (accessed on 18 June 2026).
